# Supplementary material for: Fifteenth century CE Bolivian maize reveals genetic affinities with ancient Peruvian maize
Source: eLife. 2025 Nov 18;14:RP106818. doi: 10.7554/eLife.106818 (PMC12626418; doi:10.7554/eLife.106818)
Supplement: Figure 6—source data 1. [file elife-106818-fig6-data1.pdf]

Supplementary Dataset 1. Modern Sample information.

Number	ID	IDname	Publication	Grzybowski et al.2023_ID	Organism
1	USA_HotevillaArizona	mUSA1	Wang et al. 2017	RIMMA0415	Zea mays
2	USA_AcomaPueblo	mUSA2	Wang et al. 2017	RIMMA0383	Zea mays
3	USA_JemezPueblo	mUSA3	Wang et al. 2017	RIMMA0387	Zea mays
4	USA_SanLorenzoPueblo	mUSA4	Wang et al. 2017	RIMMA0384	Zea mays
5	USA_TesuquePueblo	mUSA5	Wang et al. 2017	RIMMA1012	Zea mays
6	USA_TaosPueblo	mUSA6	Wang et al. 2017	RIMMA0385	Zea mays
7	Mexico_Jalisco	mMexico1	Wang et al. 2017	RIMMA0623	Zea mays
8	Mexico_Zacatecas	mMexico2	Wang et al. 2017	RIMMA0677	Zea mays
9	Mexico	mMexico3	Wang et al. 2017	RIMMA0672	Zea mays
10	Mexico_LaConcordiaGuerrero	mMexico4	Wang et al. 2017	RIMMA1010	Zea mays
11	Mexico_Puebla1	mMexico5	Wang et al. 2017	RIMMA0421	Zea mays
12	Mexico_Puebla2	mMexico6	Wang et al. 2017	RIMMA0626	Zea mays
13	Mexico_Puebla3	mMexico7	Wang et al. 2017	RIMMA0625	Zea mays
14	Mexico_Oaxac	mMexico8	Wang et al. 2017	RIMMA0733	Zea mays
15	Mexico_Chiapas	mMexico9	Wang et al. 2017	RIMMA0409	Zea mays
16	Guatemala_SanMarcos1	mGuatema1	Wang et al. 2017	RIMMA1007	Zea mays
17	Guatemala_SanMarcos2	mGuatema2	Wang et al. 2017	RIMMA0670	Zea mays
18	Guatemala_Totonicapan	mGuatema3	Wang et al. 2017	RIMMA1008	Zea mays
19	Guatemala	mGuatema4	Wang et al. 2017	RIMMA0720	Zea mays
20	Mexico_Yucatan	mMexico10	Wang et al. 2017	RIMMA0703	Zea mays
21	Ecuador1	mEcuador1	Wang et al. 2017	RIMMA0665	Zea mays
22	Ecuador2	mEcuador2	Wang et al. 2017	RIMMA0662	Zea mays
23	Peru_Ancash1	mPeru13	Wang et al. 2017	RIMMA0438	Zea mays
24	Colombia_Choco	mColombia1	Wang et al. 2017	RIMMA0395	Zea mays
25	Peru_Ancash2	mPeru17	Wang et al. 2017	RIMMA0468	Zea mays
26	Colombia_Magdalena1	mColombia2	Wang et al. 2017	RIMMA0398	Zea mays
27	Colombia_Caldas	mColombia3	Wang et al. 2017	RIMMA0390	Zea mays
28	Colombia_Caqueta	mColombia4	Wang et al. 2017	RIMMA0392	Zea mays
29	Colombia_Cordoba	mColombia5	Wang et al. 2017	RIMMA0393	Zea mays
30	Colombia_Magdalena2	mColombia6	Wang et al. 2017	RIMMA0399	Zea mays
31	Peru_Apurimac	mPeru3	Wang et al. 2017	RIMMA0466	Zea mays

32	Peru_Lalibertad1	mPeru4	Kistler et al. 2018	C1_Kistler	Zea mays
33	Peru_Lima	mPeru5	Kistler et al. 2018	C10_Kistler	Zea mays
34	Peru_Apurimac1	mPeru6	Kistler et al. 2018	C11_Kistler	Zea mays
35	Peru_Ica	mPeru7	Kistler et al. 2018	C12_Kistler	Zea mays
36	Peru_Arequipa	mPeru8	Kistler et al. 2018	C13_Kistler	Zea mays
37	Peru_Lambayeque	mPeru9	Kistler et al. 2018	C14_Kistler	Zea mays
38	Peru_Ayacucho	mPeru10	Kistler et al. 2018	C15_Kistler	Zea mays
39	Peru_Amazonas	mPeru11	Kistler et al. 2018	C17_Kistler	Zea mays
40	Peru_Lalibertad2	mPeru12	Kistler et al. 2018	C18_Kistler	Zea mays
41	Peru_Ancash3	mPeru1	Kistler et al. 2018	C19_Kistler	Zea mays
42	Peru_Apurimac2	mPeru14	Kistler et al. 2018	C20_Kistler	Zea mays
43	Peru_SanMartin	mPeru15	Kistler et al. 2018	C21_Kistler	Zea mays
44	Peru_Apurimac3	mPeru16	Kistler et al. 2018	C22_Kistler	Zea mays
45	Peru_Ancash4	mPeru2	Kistler et al. 2018	C23_Kistler	Zea mays
46	Peru_Puno	mPeru18	Kistler et al. 2018	C26_Kistler	Zea mays
47	Brazil_Acre1	mBrazil1	Kistler et al. 2018	C27_Kistler	Zea mays
48	Brazil_Rondonia1	mBrazil2	Kistler et al. 2018	C28_Kistler	Zea mays
49	Brazil_Parana	mBrazil3	Kistler et al. 2018	C29_Kistler	Zea mays
50	Peru_Apurimac4	mPeru19	Kistler et al. 2018	C3_Kistler	Zea mays
51	Paraguay	mParaguay	Kistler et al. 2018	C30_Kistler	Zea mays
52	Brazil_Para1	mBrazil4	Kistler et al. 2018	C31_Kistler	Zea mays
53	Brazil_SaoPaulo1	mBrazil5	Kistler et al. 2018	C32_Kistler	Zea mays
54	Brazil_Para2	mBrazil6	Kistler et al. 2018	C33_Kistler	Zea mays
55	Brazil_Altamira	mBrazil7	Kistler et al. 2018	C34_Kistler	Zea mays
56	Brazil_Maranhao	mBrazil8	Kistler et al. 2018	C35_Kistler	Zea mays
57	Brazil_Acre2	mBrazil9	Kistler et al. 2018	C36_Kistler	Zea mays
58	Brazil_SaoPaulo2	mBrazil10	Kistler et al. 2018	C38_Kistler	Zea mays
59	Brazil_Rondonia2	mBrazil11	Kistler et al. 2018	C39_Kistler	Zea mays
60	Brazil_Acre3	mBrazil12	Kistler et al. 2018	C40_Kistler	Zea mays
61	Brazil_MatoGrossoSul	mBrazil13	Kistler et al. 2018	C42_Kistler	Zea mays
62	Brazil_Para3	mBrazil14	Kistler et al. 2018	C43_Kistler	Zea mays
63	Brazil_Rondonia3	mBrazil15	Kistler et al. 2018	C44_Kistler	Zea mays
64	Brazil_MatoGrosso	mBrazil16	Kistler et al. 2018	C45_Kistler	Zea mays

65	Brazil_Goias	mBrazil17	Kistler et al. 2018	C46_Kistler	Zea mays
66	Brazil_Roraima4	mBrazil18	Kistler et al. 2018	C47_Kistler	Zea mays
67	Brazil_Roraima5	mBrazil19	Kistler et al. 2018	C48_Kistler	Zea mays
68	Peru_Junin	mPeru20	Kistler et al. 2018	C5_Kistler	Zea mays
69	Peru_Apurimac5	mPeru21	Kistler et al. 2018	C6_Kistler	Zea mays
70	Peru_MadredeDios	mPeru22	Kistler et al. 2018	C8_Kistler	Zea mays
71	Peru_Apurimac6	mPeru23	Kistler et al. 2018	C9_Kistler	Zea mays
72	Parviglumis_1	Parviglumis1	Chen et al., 2022	5A10	Z. parviglumis
73	Parviglumis_2	Parviglumis2	Chen et al., 2022	5A11	Z. parviglumis
74	Parviglumis_3	Parviglumis3	Chen et al., 2022	5A8	Z. parviglumis
75	Parviglumis_4	Parviglumis4	Chen et al., 2022	5A9	Z. parviglumis
76	Parviglumis_5	Parviglumis5	Chen et al., 2022	5B2	Z. parviglumis
77	Parviglumis_6	Parviglumis6	Chen et al., 2022	5B3	Z. parviglumis
78	Parviglumis_7	Parviglumis7	Chen et al., 2022	5B4	Z. parviglumis
79	Parviglumis_8	Parviglumis8	Chen et al., 2022	5B5	Z. parviglumis
80	Parviglumis_9	Parviglumis9	Chen et al., 2022	5B6	Z. parviglumis
81	Parviglumis_10	Parviglumis10	Chen et al., 2022	5C1	Z. parviglumis
82	Parviglumis_11	Parviglumis11	Chen et al., 2022	5C10	Z. parviglumis
83	Parviglumis_12	Parviglumis12	Chen et al., 2022	5C11	Z. parviglumis
84	Parviglumis_13	Parviglumis13	Chen et al., 2022	5C12	Z. parviglumis
85	Parviglumis_14	Parviglumis14	Chen et al., 2022	5C2	Z. parviglumis
86	Parviglumis_15	Parviglumis15	Chen et al., 2022	5C3	Z. parviglumis
87	Parviglumis_16	Parviglumis16	Chen et al., 2022	5C4	Z. parviglumis
88	Parviglumis_17	Parviglumis17	Chen et al., 2022	5C5	Z. parviglumis
89	Parviglumis_18	Parviglumis18	Chen et al., 2022	5C6	Z. parviglumis
90	Parviglumis_19	Parviglumis19	Chen et al., 2022	5C7	Z. parviglumis
91	Parviglumis_20	Parviglumis20	Chen et al., 2022	5C8	Z. parviglumis
92	Parviglumis_21	Parviglumis21	Chen et al., 2022	5C9	Z. parviglumis
93	Parviglumis_22	Parviglumis22	Chen et al., 2022	5D10	Z. parviglumis
94	Parviglumis_23	Parviglumis23	Chen et al., 2022	5D11	Z. parviglumis
95	Parviglumis_24	Parviglumis24	Chen et al., 2022	5D12	Z. parviglumis
96	Parviglumis_25	Parviglumis25	Chen et al., 2022	5D4	Z. parviglumis
97	Parviglumis_26	Parviglumis26	Chen et al., 2022	5E1	Z. parviglumis

98	Parviglumis_27	Parviglumis27	Chen et al., 2022	5E2	Z. parviglumis
99	Parviglumis_28	Parviglumis28	Chen et al., 2022	5E3	Z. parviglumis
100	Parviglumis_29	Parviglumis29	Chen et al., 2022	5E5	Z. parviglumis
101	Parviglumis_30	Parviglumis30	Chen et al., 2022	5E6	Z. parviglumis
102	Parviglumis_31	Parviglumis31	Chen et al., 2022	5F11	Z. parviglumis
103	Parviglumis_32	Parviglumis32	Chen et al., 2022	5F8	Z. parviglumis
104	Parviglumis_33	Parviglumis33	Chen et al., 2022	5F9	Z. parviglumis
105	Parviglumis_34	Parviglumis34	Chen et al., 2022	5H10	Z. parviglumis
106	Parviglumis_35	Parviglumis35	Chen et al., 2022	5H11	Z. parviglumis
107	Parviglumis_36	Parviglumis36	Chen et al., 2022	5H12	Z. parviglumis
108	Parviglumis_37	Parviglumis37	Chen et al., 2022	5H7	Z. parviglumis
109	Parviglumis_38	Parviglumis38	Chen et al., 2022	5H8	Z. parviglumis
110	Parviglumis_39	Parviglumis39	Chen et al., 2022	5H9	Z. parviglumis
111	Parviglumis_40	Parviglumis40	Chen et al., 2022	6C10	Z. parviglumis
112	Parviglumis_41	Parviglumis41	Chen et al., 2022	6C11	Z. parviglumis
113	Parviglumis_42	Parviglumis42	Chen et al., 2022	6C12	Z. parviglumis
114	Parviglumis_43	Parviglumis43	Chen et al., 2022	6D1	Z. parviglumis
115	Parviglumis_44	Parviglumis44	Chen et al., 2022	6D10	Z. parviglumis
116	Parviglumis_45	Parviglumis45	Chen et al., 2022	6D12	Z. parviglumis
117	Parviglumis_46	Parviglumis46	Chen et al., 2022	6D2	Z. parviglumis
118	Parviglumis_47	Parviglumis47	Chen et al., 2022	6D3	Z. parviglumis
119	Parviglumis_48	Parviglumis48	Chen et al., 2022	6D4	Z. parviglumis
120	Parviglumis_49	Parviglumis49	Chen et al., 2022	6D5	Z. parviglumis
121	Parviglumis_50	Parviglumis50	Chen et al., 2022	6D6	Z. parviglumis
122	Parviglumis_51	Parviglumis51	Chen et al., 2022	6D7	Z. parviglumis
123	Parviglumis_52	Parviglumis52	Chen et al., 2022	6D8	Z. parviglumis
124	Parviglumis_53	Parviglumis53	Chen et al., 2022	6D9	Z. parviglumis
125	Parviglumis_54	Parviglumis54	Chen et al., 2022	6E2	Z. parviglumis
126	Parviglumis_55	Parviglumis55	Chen et al., 2022	6E3	Z. parviglumis
127	Parviglumis_56	Parviglumis56	Chen et al., 2022	6E4	Z. parviglumis
128	Parviglumis_57	Parviglumis57	Chen et al., 2022	6E7	Z. parviglumis
129	Parviglumis_58	Parviglumis58	Chen et al., 2022	6F11	Z. parviglumis
130	Parviglumis_59	Parviglumis59	Chen et al., 2022	6F12	Z. parviglumis

131	Parviglumis_60	Parviglumis60	Chen et al., 2022	6G11	Z. parviglumis
132	Parviglumis_61	Parviglumis61	Chen et al., 2022	6G6	Z. parviglumis
133	Parviglumis_62	Parviglumis62	Chen et al., 2022	6H1	Z. parviglumis
134	Parviglumis_63	Parviglumis63	Chen et al., 2022	6H5	Z. parviglumis
135	Parviglumis_64	Parviglumis64	Chen et al., 2022	6H6	Z. parviglumis
136	Parviglumis_65	Parviglumis65	Chen et al., 2022	6H7	Z. parviglumis
137	Parviglumis_66	Parviglumis66	Chen et al., 2022	6H8	Z. parviglumis
138	Parviglumis_67	Parviglumis67	Chen et al., 2022	6H9	Z. parviglumis
139	Parviglumis_68	Parviglumis68	Chia et al. 2012	TIL01	Z. parviglumis
140	Parviglumis_69	Parviglumis69	Chia et al. 2012	TIL02	Z. parviglumis
141	Parviglumis_70	Parviglumis70	Chia et al. 2012	TIL03	Z. parviglumis
142	Parviglumis_71	Parviglumis71	Chia et al. 2012	TIL05	Z. parviglumis
143	Parviglumis_72	Parviglumis72	Chia et al. 2012	TIL06	Z. parviglumis
144	Parviglumis_73	Parviglumis73	Chia et al. 2012	TIL07	Z. parviglumis
145	Parviglumis_74	Parviglumis74	Chia et al. 2012	TIL08	Z. parviglumis
146	Parviglumis_75	Parviglumis75	Chia et al. 2012	TIL09	Z. parviglumis
147	Parviglumis_76	Parviglumis76	Chia et al. 2012	TIL10	Z. parviglumis
148	Parviglumis_77	Parviglumis77	Chia et al. 2012	TIL11	Z. parviglumis
149	Parviglumis_78	Parviglumis78	Chia et al. 2012	TIL12	Z. parviglumis
150	Parviglumis_79	Parviglumis79	Chia et al. 2012	TIL14	Z. parviglumis
151	Parviglumis_80	Parviglumis80	Chia et al. 2012	TIL15	Z. parviglumis
152	Parviglumis_81	Parviglumis81	Chia et al. 2012	TIL16	Z. parviglumis
153	Parviglumis_82	Parviglumis82	Chia et al. 2012	TIL17	Z. parviglumis
154	Parviglumis_83	Parviglumis83	Chia et al. 2012	TIL25	Z. parviglumis
155	Parviglumis_84	Parviglumis84	Unterseer et al. 2014	Teosinte	Z. parviglumis
156	Parviglumis_85	Parviglumis85	Wang et al. 2017	parviglumis_2A	Z. parviglumis
157	Parviglumis_86	Parviglumis86	Wang et al. 2017	parviglumis_2B	Z. parviglumis
158	Parviglumis_87	Parviglumis87	Wang et al. 2017	parviglumis_2C	Z. parviglumis
159	Parviglumis_88	Parviglumis88	Wang et al. 2017	parviglumis_2D	Z. parviglumis
160	Tripsacum	tripsacum	Chen et al., 2022	tripsacum	Tripsacum dactyloides
161	Zmexicana_1	Zmexicana1	Chen et al., 2022	5A1	Z. mexicana
162	Zmexicana_2	Zmexicana2	Chen et al., 2022	5A2	Z. mexicana
163	Zmexicana_3	Zmexicana3	Chen et al., 2022	5A3	Z. mexicana

164	Zmexicana_4	Zmexicana4	Chen et al., 2022	5A4	Z. mexicana
165	Zmexicana_5	Zmexicana5	Chen et al., 2022	5A5	Z. mexicana
166	Zmexicana_6	Zmexicana6	Chen et al., 2022	5A6	Z. mexicana
167	Zmexicana_7	Zmexicana7	Chen et al., 2022	5A7	Z. mexicana
168	Zmexicana_8	Zmexicana8	Chen et al., 2022	5B7	Z. mexicana
169	Zmexicana_9	Zmexicana9	Chen et al., 2022	5B8	Z. mexicana
170	Zmexicana_10	Zmexicana10	Chen et al., 2022	5B9	Z. mexicana
171	Zmexicana_11	Zmexicana11	Chen et al., 2022	5D9	Z. mexicana
172	Zmexicana_12	Zmexicana12	Chen et al., 2022	5E10	Z. mexicana
173	Zmexicana_13	Zmexicana13	Chen et al., 2022	5E11	Z. mexicana
174	Zmexicana_14	Zmexicana14	Chen et al., 2022	5E12	Z. mexicana
175	Zmexicana_15	Zmexicana15	Chen et al., 2022	5E7	Z. mexicana
176	Zmexicana_16	Zmexicana16	Chen et al., 2022	5E8	Z. mexicana
177	Zmexicana_17	Zmexicana17	Chen et al., 2022	5E9	Z. mexicana
178	Zmexicana_18	Zmexicana18	Chen et al., 2022	5F1	Z. mexicana
179	Zmexicana_19	Zmexicana19	Chen et al., 2022	5F10	Z. mexicana
180	Zmexicana_20	Zmexicana20	Chen et al., 2022	5F12	Z. mexicana
181	Zmexicana_21	Zmexicana21	Chen et al., 2022	5F2	Z. mexicana
182	Zmexicana_22	Zmexicana22	Chen et al., 2022	5F3	Z. mexicana
183	Zmexicana_23	Zmexicana23	Chen et al., 2022	5F4	Z. mexicana
184	Zmexicana_24	Zmexicana24	Chen et al., 2022	5F5	Z. mexicana
185	Zmexicana_25	Zmexicana25	Chen et al., 2022	5F6	Z. mexicana
186	Zmexicana_26	Zmexicana26	Chen et al., 2022	5F7	Z. mexicana
187	Zmexicana_27	Zmexicana27	Chen et al., 2022	5G1	Z. mexicana
188	Zmexicana_28	Zmexicana28	Chen et al., 2022	5G10	Z. mexicana
189	Zmexicana_29	Zmexicana29	Chen et al., 2022	5G12	Z. mexicana
190	Zmexicana_30	Zmexicana30	Chen et al., 2022	5G2	Z. mexicana
191	Zmexicana_31	Zmexicana31	Chen et al., 2022	5G3	Z. mexicana
192	Zmexicana_32	Zmexicana32	Chen et al., 2022	5G6	Z. mexicana
193	Zmexicana_33	Zmexicana33	Chen et al., 2022	5G7	Z. mexicana
194	Zmexicana_34	Zmexicana34	Chen et al., 2022	5G8	Z. mexicana
195	Zmexicana_35	Zmexicana35	Chen et al., 2022	5G9	Z. mexicana
196	Zmexicana_36	Zmexicana36	Chen et al., 2022	5H1	Z. mexicana

197	Zmexicana_37	Zmexicana37	Chen et al., 2022	5H2	Z. mexicana
198	Zmexicana_38	Zmexicana38	Chen et al., 2022	5H3	Z. mexicana
199	Zmexicana_39	Zmexicana39	Chen et al., 2022	5H4	Z. mexicana
200	Zmexicana_40	Zmexicana40	Chen et al., 2022	5H5	Z. mexicana
201	Zmexicana_41	Zmexicana41	Chen et al., 2022	6A1	Z. mexicana
202	Zmexicana_42	Zmexicana42	Chen et al., 2022	6A10	Z. mexicana
203	Zmexicana_43	Zmexicana43	Chen et al., 2022	6A11	Z. mexicana
204	Zmexicana_44	Zmexicana44	Chen et al., 2022	6A12	Z. mexicana
205	Zmexicana_45	Zmexicana45	Chen et al., 2022	6A2	Z. mexicana
206	Zmexicana_46	Zmexicana46	Chen et al., 2022	6A3	Z. mexicana
207	Zmexicana_47	Zmexicana47	Chen et al., 2022	6A4	Z. mexicana
208	Zmexicana_48	Zmexicana48	Chen et al., 2022	6A5	Z. mexicana
209	Zmexicana_49	Zmexicana49	Chen et al., 2022	6A6	Z. mexicana
210	Zmexicana_50	Zmexicana50	Chen et al., 2022	6A7	Z. mexicana
211	Zmexicana_51	Zmexicana51	Chen et al., 2022	6A8	Z. mexicana
212	Zmexicana_52	Zmexicana52	Chen et al., 2022	6A9	Z. mexicana
213	Zmexicana_53	Zmexicana53	Chen et al., 2022	6B10	Z. mexicana
214	Zmexicana_54	Zmexicana54	Chen et al., 2022	6B11	Z. mexicana
215	Zmexicana_55	Zmexicana55	Chen et al., 2022	6B12	Z. mexicana
216	Zmexicana_56	Zmexicana56	Chen et al., 2022	6B2	Z. mexicana
217	Zmexicana_57	Zmexicana57	Chen et al., 2022	6B3	Z. mexicana
218	Zmexicana_58	Zmexicana58	Chen et al., 2022	6B4	Z. mexicana
219	Zmexicana_59	Zmexicana59	Chen et al., 2022	6B5	Z. mexicana
220	Zmexicana_60	Zmexicana60	Chen et al., 2022	6B6	Z. mexicana
221	Zmexicana_61	Zmexicana61	Chen et al., 2022	6B7	Z. mexicana
222	Zmexicana_62	Zmexicana62	Chen et al., 2022	6B8	Z. mexicana
223	Zmexicana_63	Zmexicana63	Chen et al., 2022	6B9	Z. mexicana
224	Zmexicana_64	Zmexicana64	Chen et al., 2022	6C1	Z. mexicana
225	Zmexicana_65	Zmexicana65	Chen et al., 2022	6C2	Z. mexicana
226	Zmexicana_66	Zmexicana66	Chen et al., 2022	6C3	Z. mexicana
227	Zmexicana_67	Zmexicana67	Chen et al., 2022	6C4	Z. mexicana
228	Zmexicana_68	Zmexicana68	Chen et al., 2022	6C5	Z. mexicana
229	Zmexicana_69	Zmexicana69	Chen et al., 2022	6C6	Z. mexicana

230	Zmexicana_70	Zmexicana70	Chen et al., 2022	6C7	Z. mexicana
231	Zmexicana_71	Zmexicana71	Chen et al., 2022	6C8	Z. mexicana
232	Zmexicana_72	Zmexicana72	Chen et al., 2022	6C9	Z. mexicana
233	Zmexicana_73	Zmexicana73	Chen et al., 2022	6D11	Z. mexicana
234	Zmexicana_74	Zmexicana74	Chen et al., 2022	6E1	Z. mexicana
235	Zmexicana_75	Zmexicana75	Chen et al., 2022	6E10	Z. mexicana
236	Zmexicana_76	Zmexicana76	Chen et al., 2022	6E5	Z. mexicana
237	Zmexicana_77	Zmexicana77	Chen et al., 2022	6E9	Z. mexicana
238	Zmexicana_78	Zmexicana78	Chen et al., 2022	6H2	Z. mexicana
239	Zmexicana_79	Zmexicana79	Chen et al., 2022	6H4	Z. mexicana
240	SweetCorn_1	SweetCorn1	Grzybowski et al. 2023	4554_INBRED	Z. mays Sweet corn
241	SweetCorn_2	SweetCorn2	Grzybowski et al. 2023	80-2	Z. mays Sweet corn
242	SweetCorn_3	SweetCorn3	Grzybowski et al. 2023	C15	Z. mays Sweet corn
243	SweetCorn_4	SweetCorn4	Grzybowski et al. 2023	C42	Z. mays Sweet corn
244	SweetCorn_5	SweetCorn5	Grzybowski et al. 2023	C68	Z. mays Sweet corn
245	SweetCorn_6	SweetCorn6	Qiu et al. 2021	CA-4	Z. mays Sweet corn
246	SweetCorn_7	SweetCorn7	Grzybowski et al. 2023	CL17	Z. mays Sweet corn
247	SweetCorn_8	SweetCorn8	Grzybowski et al. 2023	CL27	Z. mays Sweet corn
248	SweetCorn_9	SweetCorn9	Grzybowski et al. 2023	CO245	Z. mays Sweet corn
249	SweetCorn_10	SweetCorn10	Bukowski et al. 2018	CO255	Z. mays Sweet corn
250	SweetCorn_11	SweetCorn11	Bukowski et al. 2018	EP1	Z. mays Sweet corn
251	SweetCorn_12	SweetCorn12	Unterseer et al. 2014	F2	Z. mays Sweet corn
252	SweetCorn_13	SweetCorn13	Bukowski et al. 2018	F7	Z. mays Sweet corn
253	SweetCorn_14	SweetCorn14	Qiu et al. 2021	FC46	Z. mays Sweet corn
254	SweetCorn_15	SweetCorn15	Grzybowski et al. 2023	G22_T122	Z. mays Sweet corn
255	SweetCorn_16	SweetCorn16	Grzybowski et al. 2023	G3_T5a	Z. mays Sweet corn
256	SweetCorn_17	SweetCorn17	Qiu et al. 2021	IA2132	Z. mays Sweet corn
257	SweetCorn_18	SweetCorn18	Grzybowski et al. 2023	Ia453	Z. mays Sweet corn
258	SweetCorn_19	SweetCorn19	Bukowski et al. 2018	Ia5125	Z. mays Sweet corn
259	SweetCorn_20	SweetCorn20	Grzybowski et al. 2023	Ia5125B	Z. mays Sweet corn
260	SweetCorn_21	SweetCorn21	Bukowski et al. 2018	Il101	Z. mays Sweet corn
261	SweetCorn_22	SweetCorn22	Bukowski et al. 2018	Il14H	Z. mays Sweet corn
262	SweetCorn_23	SweetCorn23	Grzybowski et al. 2023	Il778d	Z. mays Sweet corn

263	SweetCorn_24	SweetCorn24	Grzybowski et al. 2023	Il_101T	Z. mays Sweet corn
264	SweetCorn_25	SweetCorn25	Grzybowski et al. 2023	NO._380	Z. mays Sweet corn
265	SweetCorn_26	SweetCorn26	Bukowski et al. 2018	P39	Z. mays Sweet corn
266	SweetCorn_27	SweetCorn27	Grzybowski et al. 2023	PHDD6	Z. mays Sweet corn
267	SweetCorn_28	SweetCorn28	Grzybowski et al. 2023	PHEM7	Z. mays Sweet corn
268	SweetCorn_29	SweetCorn29	Grzybowski et al. 2023	PHGG7	Z. mays Sweet corn
269	SweetCorn_30	SweetCorn30	Grzybowski et al. 2023	S_56	Z. mays Sweet corn
270	SweetCorn_31	SweetCorn31	Grzybowski et al. 2023	T146	Z. mays Sweet corn
271	SweetCorn_32	SweetCorn32	Qiu et al. 2021	T242	Z. mays Sweet corn
272	SweetCorn_33	SweetCorn33	Grzybowski et al. 2023	T9	Z. mays Sweet corn
273	SweetCorn_34	SweetCorn34	Grzybowski et al. 2023	U_123	Z. mays Sweet corn
274	SweetCorn_35	SweetCorn35	Bukowski et al. 2018	i1677a	Z. mays Sweet corn
275	Ztropical_1	Ztropical1	Grzybowski et al. 2023	4F-306_108	Z. mays Tropical
276	Ztropical_2	Ztropical2	Qiu et al. 2021	4F-35_BK	Z. mays Tropical
277	Ztropical_3	Ztropical3	Qiu et al. 2021	A272	Z. mays Tropical
278	Ztropical_4	Ztropical4	Grzybowski et al. 2023	A3G-3-3-1-313	Z. mays Tropical
279	Ztropical_5	Ztropical5	Bukowski et al. 2018	CML10	Z. mays Tropical
280	Ztropical_6	Ztropical6	Bukowski et al. 2018	CML11	Z. mays Tropical
281	Ztropical_7	Ztropical7	Bukowski et al. 2018	CML14	Z. mays Tropical
282	Ztropical_8	Ztropical8	Bukowski et al. 2018	CML157Q	Z. mays Tropical
283	Ztropical_9	Ztropical9	Bukowski et al. 2018	CML158Q	Z. mays Tropical
284	Ztropical_10	Ztropical10	Bukowski et al. 2018	CML238	Z. mays Tropical
285	Ztropical_11	Ztropical11	Bukowski et al. 2018	CML258	Z. mays Tropical
286	Ztropical_12	Ztropical12	Bukowski et al. 2018	CML261	Z. mays Tropical
287	Ztropical_13	Ztropical13	Bukowski et al. 2018	CML281	Z. mays Tropical
288	Ztropical_14	Ztropical14	Bukowski et al. 2018	CML311	Z. mays Tropical
289	Ztropical_15	Ztropical15	Bukowski et al. 2018	CML314	Z. mays Tropical
290	Ztropical_16	Ztropical16	Bukowski et al. 2018	CML321	Z. mays Tropical
291	Ztropical_17	Ztropical17	Bukowski et al. 2018	CML331	Z. mays Tropical
292	Ztropical_18	Ztropical18	Bukowski et al. 2018	CML332	Z. mays Tropical
293	Ztropical_19	Ztropical19	Bukowski et al. 2018	CML333	Z. mays Tropical
294	Ztropical_20	Ztropical20	Bukowski et al. 2018	CML341	Z. mays Tropical
295	Ztropical_21	Ztropical21	Bukowski et al. 2018	CML38	Z. mays Tropical

296	Ztropical_22	Ztropical22	Bukowski et al. 2018	CML45	Z. mays Tropical
297	Ztropical_23	Ztropical23	Bukowski et al. 2018	CML5	Z. mays Tropical
298	Ztropical_24	Ztropical24	Bukowski et al. 2018	CML61	Z. mays Tropical
299	Ztropical_25	Ztropical25	Bukowski et al. 2018	CML69	Z. mays Tropical
300	Ztropical_26	Ztropical26	Bukowski et al. 2018	CML_108	Z. mays Tropical
301	Ztropical_27	Ztropical27	Bukowski et al. 2018	CML_154Q	Z. mays Tropical
302	Ztropical_28	Ztropical28	Bukowski et al. 2018	CML_218	Z. mays Tropical
303	Ztropical_29	Ztropical29	Bukowski et al. 2018	CML_220	Z. mays Tropical
304	Ztropical_30	Ztropical30	Bukowski et al. 2018	CML_228	Z. mays Tropical
305	Ztropical_31	Ztropical31	Bukowski et al. 2018	CML_247	Z. mays Tropical
306	Ztropical_32	Ztropical32	Bukowski et al. 2018	CML_254	Z. mays Tropical
307	Ztropical_33	Ztropical33	Bukowski et al. 2018	CML_264	Z. mays Tropical
308	Ztropical_34	Ztropical34	Bukowski et al. 2018	CML_277	Z. mays Tropical
309	Ztropical_35	Ztropical35	Bukowski et al. 2018	CML_287	Z. mays Tropical
310	Ztropical_36	Ztropical36	Bukowski et al. 2018	CML_322	Z. mays Tropical
311	Ztropical_37	Ztropical37	Bukowski et al. 2018	CML_323	Z. mays Tropical
312	Ztropical_38	Ztropical38	Grzybowski et al. 2023	CML_395	Z. mays Tropical
313	Ztropical_39	Ztropical39	Bukowski et al. 2018	CML_52	Z. mays Tropical
314	Ztropical_40	Ztropical40	Bukowski et al. 2018	CML_91	Z. mays Tropical
315	Ztropical_41	Ztropical41	Qiu et al. 2021	F2834T	Z. mays Tropical
316	Ztropical_42	Ztropical42	Grzybowski et al. 2023	Hi28	Z. mays Tropical
317	Ztropical_43	Ztropical43	Qiu et al. 2021	Huanyao	Z. mays Tropical
318	Ztropical_44	Ztropical44	Grzybowski et al. 2023	INBRED_100	Z. mays Tropical
319	Ztropical_45	Ztropical45	Grzybowski et al. 2023	INBRED_109	Z. mays Tropical
320	Ztropical_46	Ztropical46	Grzybowski et al. 2023	INBRED_2-687	Z. mays Tropical
321	Ztropical_47	Ztropical47	Grzybowski et al. 2023	INBRED_305	Z. mays Tropical
322	Ztropical_48	Ztropical48	Grzybowski et al. 2023	INBRED_309	Z. mays Tropical
323	Ztropical_49	Ztropical49	Bukowski et al. 2018	Ki11	Z. mays Tropical
324	Ztropical_50	Ztropical50	Bukowski et al. 2018	Ki14	Z. mays Tropical
325	Ztropical_51	Ztropical51	Bukowski et al. 2018	Ki2021	Z. mays Tropical
326	Ztropical_52	Ztropical52	Bukowski et al. 2018	Ki3	Z. mays Tropical
327	Ztropical_53	Ztropical53	Bukowski et al. 2018	Ki43	Z. mays Tropical
328	Ztropical_54	Ztropical54	Bukowski et al. 2018	Ki44	Z. mays Tropical

329	Ztropical_55	Ztropical55	Wang et al. 2020	MO18W	Z. mays Tropical
330	Ztropical_56	Ztropical56	Bukowski et al. 2018	NC264	Z. mays Tropical
331	Ztropical_57	Ztropical57	Bukowski et al. 2018	NC296	Z. mays Tropical
332	Ztropical_58	Ztropical58	Bukowski et al. 2018	NC296A	Z. mays Tropical
333	Ztropical_59	Ztropical59	Bukowski et al. 2018	NC298	Z. mays Tropical
334	Ztropical_60	Ztropical60	Bukowski et al. 2018	NC300	Z. mays Tropical
335	Ztropical_61	Ztropical61	Bukowski et al. 2018	NC302	Z. mays Tropical
336	Ztropical_62	Ztropical62	Bukowski et al. 2018	NC304	Z. mays Tropical
337	Ztropical_63	Ztropical63	Bukowski et al. 2018	NC320	Z. mays Tropical
338	Ztropical_64	Ztropical64	Bukowski et al. 2018	NC336	Z. mays Tropical
339	Ztropical_65	Ztropical65	Bukowski et al. 2018	NC338	Z. mays Tropical
340	Ztropical_66	Ztropical66	Bukowski et al. 2018	NC340	Z. mays Tropical
341	Ztropical_67	Ztropical67	Bukowski et al. 2018	NC346	Z. mays Tropical
342	Ztropical_68	Ztropical68	Bukowski et al. 2018	NC348	Z. mays Tropical
343	Ztropical_69	Ztropical69	Bukowski et al. 2018	NC350	Z. mays Tropical
344	Ztropical_70	Ztropical70	Bukowski et al. 2018	NC352	Z. mays Tropical
345	Ztropical_71	Ztropical71	Bukowski et al. 2018	NC354	Z. mays Tropical
346	Ztropical_72	Ztropical72	Bukowski et al. 2018	NC356	Z. mays Tropical
347	Ztropical_73	Ztropical73	Bukowski et al. 2018	NC358	Z. mays Tropical
348	Ztropical_74	Ztropical74	Qiu et al. 2021	NY6371	Z. mays Tropical
349	Ztropical_75	Ztropical75	Bukowski et al. 2018	Tx601	Z. mays Tropical
350	Ztropical_76	Ztropical76	Bukowski et al. 2018	Tzi10	Z. mays Tropical
351	Ztropical_77	Ztropical77	Bukowski et al. 2018	Tzi11	Z. mays Tropical
352	Ztropical_78	Ztropical78	Bukowski et al. 2018	Tzi18	Z. mays Tropical
353	Ztropical_79	Ztropical79	Bukowski et al. 2018	Tzi8	Z. mays Tropical
354	Ztropical_80	Ztropical80	Bukowski et al. 2018	Tzi9	Z. mays Tropical
355	Ztropical_81	Ztropical81	Qiu et al. 2021	W803G	Z. mays Tropical
356	Ztropical_82	Ztropical82	Bukowski et al. 2018	WIL500	Z. mays Tropical
357	Ztropical_83	Ztropical83	Grzybowski et al. 2023	YANG	Z. mays Tropical
358	Ztropical_84	Ztropical84	Grzybowski et al. 2023	YE-CHI-HUNG	Z. mays Tropical
359	Ztropical_85	Ztropical85	Grzybowski et al. 2023	YELLOW_3-4	Z. mays Tropical
360	Ztropical_86	Ztropical86	Grzybowski et al. 2023	YE_4	Z. mays Tropical
361	Europe_1	Europe1	Unterseer et al. 2014	CH10	Z. mays Europe

362	Europe_2	Europe2	Unterseer et al. 2014	D06	Z. mays Europe
363	Europe_3	Europe3	Unterseer et al. 2014	D09	Z. mays Europe
364	Europe_4	Europe4	Unterseer et al. 2014	D152	Z. mays Europe
365	Europe_5	Europe5	Unterseer et al. 2014	DK105	Z. mays Europe
366	Europe_6	Europe6	Unterseer et al. 2014	EC169	Z. mays Europe
367	Europe_7	Europe7	Unterseer et al. 2014	EC49A	Z. mays Europe
368	Europe_8	Europe8	Unterseer et al. 2014	EP44	Z. mays Europe
369	Europe_9	Europe9	Unterseer et al. 2014	EZ5	Z. mays Europe
370	Europe_10	Europe10	Unterseer et al. 2014	F03802	Z. mays Europe
371	Europe_11	Europe11	Unterseer et al. 2014	F252	Z. mays Europe
372	Europe_12	Europe12	Unterseer et al. 2014	F283	Z. mays Europe
373	Europe_13	Europe13	Unterseer et al. 2014	F353	Z. mays Europe
374	Europe_14	Europe14	Unterseer et al. 2014	F618	Z. mays Europe
375	Europe_15	Europe15	Unterseer et al. 2014	F64	Z. mays Europe
376	Europe_16	Europe16	Unterseer et al. 2014	F98902	Z. mays Europe
377	Europe_17	Europe17	Unterseer et al. 2014	FF0721H-7	Z. mays Europe
378	Europe_18	Europe18	Unterseer et al. 2014	Lo11	Z. mays Europe
379	Europe_19	Europe19	Grzybowski et al. 2023	S018693	Z. mays Europe
380	Europe_20	Europe20	Grzybowski et al. 2023	S03198	Z. mays Europe
381	Europe_21	Europe21	Grzybowski et al. 2023	S160	Z. mays Europe
382	Europe_22	Europe22	Grzybowski et al. 2023	S245	Z. mays Europe
383	Europe_23	Europe23	Grzybowski et al. 2023	S25	Z. mays Europe
384	Europe_24	Europe24	Grzybowski et al. 2023	S311	Z. mays Europe
385	Europe_25	Europe25	Grzybowski et al. 2023	S336A	Z. mays Europe
386	Europe_26	Europe26	Grzybowski et al. 2023	S50676	Z. mays Europe
387	Europe_27	Europe27	Grzybowski et al. 2023	S61328	Z. mays Europe
388	Europe_28	Europe28	Grzybowski et al. 2023	S68911	Z. mays Europe
389	Europe_29	Europe29	Grzybowski et al. 2023	S84854	Z. mays Europe
390	Europe_30	Europe30	Unterseer et al. 2014	UH006	Z. mays Europe
391	Europe_31	Europe31	Unterseer et al. 2014	UH007	Z. mays Europe
392	Europe_32	Europe32	Unterseer et al. 2014	UH009	Z. mays Europe
393	Europe_33	Europe33	Unterseer et al. 2014	UH250	Z. mays Europe
394	Europe_34	Europe34	Unterseer et al. 2014	UH304	Z. mays Europe
