# Supplementary material for: Fifteenth century CE Bolivian maize reveals genetic affinities with ancient Peruvian maize
Source: eLife. 2025 Nov 18;14:RP106818. doi: 10.7554/eLife.106818 (PMC12626418; doi:10.7554/eLife.106818)
Supplement: Supplementary file 1. — (A) Geographical information of the archaeological maize samples used in this study. (B) Paleogenomic characterization of archaeological Bolivian maize sequence samples with six libraries. (C) The percentage of genomic sites covered at variable depths in the archaeological Bolivian maize (aBM) sample 766 (aBMComb_rm5nt.rmDR.bam). [file elife-106818-supp1.pdf]

Table S1. Geographical information of the archaeological maize samples used in this study.

ID	Country	Age (BP)	Location	Latitude	Longitude	Altitude (m above sea level)
Arica4	Chile	990 +/- 30	Arica, Chile, coastal	18.47S	70.32W	coastal
Arica5	Chile	780 +/- 30	Arica, Chile, coastal	18.47S	70.32W	coastal
Z2	Brazil	570 +- 60	Brazil:Peruacu Valley, Boquete cave	15S	44W	700
Z6	Brazil	630+- 60	Brazil:Peruacu Valley, Lapa de Hora	15S	44W	700
Z61	Peru	800 +/- 30	Peru:Ancash	8.531S	78.341W	200
Z64	Peru	630 +/- 30	Peru:Ancash	9.064S	77.564W	3990
Z65	Peru	970 +/- 30	Peru:Inca	14.433S	75.342W	220
Z66	Argentina	1010 +/- 30	Argentina:Catamerca	27.342S	66.550W	2465
Z67	Argentina	100 +/- 30	Argentina:Jujuy	23.240S	66.211W	3700
EG84	Honduras	1870 – 1740	El Gigante rock shelter	14.22N	88.06W	NA
EG85	Honduras	2300 – 2070	El Gigante rock shelter	14.22N	88.06W	NA
EG90	Honduras	2300 – 2120	El Gigante rock shelter	14.22N	88.06W	NA
Tehua162	Mexico	5310	Mexico: Tehuacan, Puebla	18.46N	97.39W	NA
SM3	Mexico	4190 +/- 30	Mexico: San Marcos cave, Tehuacan Valley	17.48N	97.03W	NA
SM5	Mexico	4190 +/- 30	Mexico: San Marcos cave, Tehuacan Valley	17.48N	97.03W	NA
SM10	Mexico	4240 +/- 30	Mexico: San Marcos cave, Tehuacan Valley	17.48N	97.03W	NA

* The latitude and longitude information for SM3 and SM10 were determined based on the Tehuacan Valley location as provided in 'Myxomycetes associated with dryland ecosystems of the Tehuacán-Cuicatlán Valley Biosphere Reserve, Mexico."

Table S2. Paleogenetic characterization of archaeological Bolivian maize sequence samples with six libraries.

Sample	QC-passed reads	Total number of read mapped	Read mapped (%)	Mean Coverage	Std Coverage
aBMComb_rm5nt.rmDR.bam	299093147	33846381	11.32%	0.7667X	3.027X
Bolivian_Maize_1_ATCACG_L001_rm5nt.rmDR.bam	56128831	6402435	11.41%	0.1434X	0.6598X
Bolivian_Maize_2_CGATGT_L001_rm5nt.rmDR.bam	67115071	8050825	12.00%	0.1815X	0.7718X
Bolivian_Maize_3_TTAGGC_L001_rm5nt.rmDR.bam	53043360	5998148	11.31%	0.1399X	0.6726X
Bolivian_Maize_4_TGACCA_L001_rm5nt.rmDR.bam	10941693	1579877	14.44%	0.0371X	0.2633X
Bolivian_Maize_5_ACAGTG_L001_rm5nt.rmDR.bam	10694963	1652909	15.46%	0.0361X	0.2482X
Bolivian_Maize_6_GCCAAT_L001_rm5nt.rmDR.bam	101169229	10162187	10.04%	0.2287X	0.9215X

Table S3. The percentage of genomic sites covered at variable depths in the archaeological Bolivian maize (aBM) sample. (aBMComb_rm5nt.rmDR.bam)

Depth	aBM_Combbam
1X	27.75%
2X	14.14%
3X	8.27%
4X	5.18%
5X	3.41%
6X	2.35%
7X	1.7%
8X	1.28%
9X	1%
>=10X	9.78%
