# Supplementary material for: Fifteenth century CE Bolivian maize reveals genetic affinities with ancient Peruvian maize
Source: eLife. 2025 Nov 18;14:RP106818. doi: 10.7554/eLife.106818 (PMC12626418; doi:10.7554/eLife.106818)
Supplement: Source data 1. [file elife-106818-data1.pdf]

Supplementary Dataset 2. Known SNPs related traits.

SNPs	aBM_frq	ancient_frq	Type	Gene_name	Biologoical_process	SNPs and traits
3_5721506	G:1 A:0	G:-nan A:-nan	TYPE=splice_donor_variant&intron_variant;EFFECT=HIGH;GENEMODEL=Zm00001eb120960	bhlh70	GO:0006355: regulation of transcription, DNA-templated	NA
3_13134097	G:1 A:0	G:-nan A:-nan	TYPE=stop_gained;EFFECT=HIGH;GENEMODEL=Zm00001eb123120	NA	GO:0006470: protein dephosphorylation	transcript SNP chromosome position structure trait 3:13183499; chr3; 13132965; Flanking region internode length below ear
4_76831415	A:1 G:0	A:-nan G:-nan	TYPE=stop_lost;EFFECT=HIGH;GENEMODEL=Zm00001eb178600	NA	GO:0006508: proteolysis	transcript SNP chromosome position structure trait 4:73852529; chr4; 76830220; Flanking region; nodes above ear
5_128622059	A:1 T:0	A:-nan T:-nan	TYPE=stop_gained;EFFECT=HIGH;GENEMODEL=Zm00001eb236660	NA	NA	NA
