# Supplementary material for: Fifteenth century CE Bolivian maize reveals genetic affinities with ancient Peruvian maize
Source: eLife. 2025 Nov 18;14:RP106818. doi: 10.7554/eLife.106818 (PMC12626418; doi:10.7554/eLife.106818)
Supplement: Source data 3. [file elife-106818-data3.pdf]

Supplementary Dataset 4. Go term enrichment information.

Name	GO_Term	Gene_count	Gene_list
GO:0016020	membrane	8	Zm00001eb007310,Zm00001eb026360,Zm00001eb063130,Zm00001eb105710,Zm00001eb117810,Zm00001eb178330,Zm00001eb199740,Zm00001eb204470
GO:0005524	ATP binding	5	Zm00001eb024430,Zm00001eb082590,Zm00001eb167520,Zm00001eb179000,Zm00001eb209550
GO:0015031	protein transport	4	Zm00001eb007310,Zm00001eb070240,Zm00001eb178330,Zm00001eb209550
GO:0004675	transmembrane receptor protein serine/threonine kinase activity	4	Zm00001eb117810,Zm00001eb167520,Zm00001eb178330,Zm00001eb209550
GO:0016757	glycosyltransferase activity	3	Zm00001eb105710,Zm00001eb117240,Zm00001eb167520
GO:0005663	DNA replication factor C complex	3	Zm00001eb056940,Zm00001eb178330,Zm00001eb209550
GO:0003700	DNA-binding transcription factor activity	3	Zm00001eb079800,Zm00001eb167520,Zm00001eb205130
GO:0003676	nucleic acid binding	3	Zm00001eb014140,Zm00001eb024430,Zm00001eb082590
GO:0046854	phosphatidylinositol phosphate biosynthetic process	2	Zm00001eb014140,Zm00001eb204480
GO:0006914	autophagy	2	Zm00001eb060520,Zm00001eb070240
GO:0005788	endoplasmic reticulum lumen	2	Zm00001eb007310,Zm00001eb117810
GO:1901698	response to nitrogen compound	1	Zm00001eb070240
GO:0072593	reactive oxygen species metabolic process	1	Zm00001eb060520
GO:0061630	ubiquitin protein ligase activity	1	Zm00001eb178330
GO:0055046	microgametogenesis	1	Zm00001eb187340
GO:0052742	phosphatidylinositol kinase activity	1	Zm00001eb178330
GO:0051787	misfolded protein binding	1	Zm00001eb178330
GO:0051085	chaperone cofactor-dependent protein refolding	1	Zm00001eb209550
GO:0051082	unfolded protein binding	1	Zm00001eb209550
GO:0048015	phosphatidylinositol-mediated signaling	1	Zm00001eb209550
GO:0046933	proton-transporting ATP synthase activity, rotational mechanism	1	Zm00001eb178330
GO:0046872	metal ion binding	1	Zm00001eb205130
GO:0045893	positive regulation of transcription, DNA-templated	1	Zm00001eb178330
GO:0045261	proton-transporting ATP synthase complex, catalytic core F(1)	1	Zm00001eb179000
GO:0044183	protein folding chaperone	1	Zm00001eb205130
GO:0043565	sequence-specific DNA binding	1	Zm00001eb209550
GO:0042742	defense response to bacterium	1	Zm00001eb179000
GO:0042026	protein refolding	1	Zm00001eb117810
GO:0036092	phosphatidylinositol-3-phosphate biosynthetic process	1	Zm00001eb209550
GO:0034663	endoplasmic reticulum chaperone complex	1	Zm00001eb178330
GO:0034620	cellular response to unfolded protein	1	Zm00001eb209550
GO:0034272	phosphatidylinositol 3-kinase complex, class III, type II	1	Zm00001eb209550
GO:0034271	phosphatidylinositol 3-kinase complex, class III, type I	1	Zm00001eb178330
GO:0032580	Golgi cisterna membrane	1	Zm00001eb178330
GO:0031072	heat shock protein binding	1	Zm00001eb026360
GO:0030968	endoplasmic reticulum unfolded protein response	1	Zm00001eb209550
GO:0030433	ubiquitin-dependent ERAD pathway	1	Zm00001eb209550
GO:0030246	carbohydrate binding	1	Zm00001eb209550
GO:0030242	autophagy of peroxisome	1	Zm00001eb117810
GO:0022857	transmembrane transporter activity	1	Zm00001eb178330
GO:0019252	starch biosynthetic process	1	Zm00001eb199740
GO:0016787	hydrolase activity	1	Zm00001eb070240
GO:0016639	oxidoreductase activity, acting on the CH-NH2 group of donors, NAD or NADP as acceptor	1	Zm00001eb070240
GO:0016567	protein ubiquitination	1	Zm00001eb060520
GO:0016491	oxidoreductase activity	1	Zm00001eb187340
GO:0016303	1-phosphatidylinositol-3-kinase activity	1	Zm00001eb060520
GO:0016301	kinase activity	1	Zm00001eb178330
GO:0016021	integral component of membrane	1	Zm00001eb178330
GO:0010584	pollen exine formation	1	Zm00001eb036550
GO:0010468	regulation of gene expression	1	Zm00001eb063130
GO:0010073	meristem maintenance	1	Zm00001eb205130
GO:0009658	chloroplast organization	1	Zm00001eb105710
GO:0009651	response to salt stress	1	Zm00001eb079800
GO:0009507	chloroplast	1	Zm00001eb178330
GO:0008417	fucosyltransferase activity	1	Zm00001eb070240
GO:0008270	zinc ion binding	1	Zm00001eb026360
GO:0007033	vacuole organization	1	Zm00001eb187340
GO:0006995	cellular response to nitrogen starvation	1	Zm00001eb056940
GO:0006897	endocytosis	1	Zm00001eb070240
GO:0006887	exocytosis	1	Zm00001eb178330
GO:0006857	oligopeptide transport	1	Zm00001eb036550
GO:0006538	glutamate catabolic process	1	Zm00001eb199740
GO:0006520	cellular amino acid metabolic process	1	Zm00001eb060520
GO:0006486	protein glycosylation	1	Zm00001eb060520
GO:0006355	regulation of transcription, DNA-templated	1	Zm00001eb026360
GO:0006281	DNA repair	1	Zm00001eb082590
GO:0006261	DNA-dependent DNA replication	1	Zm00001eb024430
GO:0006260	DNA replication	1	Zm00001eb024430
GO:0006096	glycolytic process	1	Zm00001eb024430
GO:0005886	plasma membrane	1	Zm00001eb194260
GO:0005783	endoplasmic reticulum	1	Zm00001eb209550
GO:0005777	peroxisome	1	Zm00001eb063130
GO:0005768	endosome	1	Zm00001eb178330
GO:0005739	mitochondrion	1	Zm00001eb178330
GO:0005737	cytoplasm	1	Zm00001eb060520
GO:0005654	nucleoplasm	1	Zm00001eb024430
GO:0005634	nucleus	1	Zm00001eb205130
GO:0004672	protein kinase activity	1	Zm00001eb117810
GO:0004619	phosphoglycerate mutase activity	1	Zm00001eb117810
GO:0004354	glutamate dehydrogenase (NADP+) activity	1	Zm00001eb194260
GO:0004352	glutamate dehydrogenase (NAD+) activity	1	Zm00001eb060520
GO:0003729	mRNA binding	1	Zm00001eb060520
GO:0003724	RNA helicase activity	1	Zm00001eb205130
GO:0003723	RNA binding	1	Zm00001eb167520
GO:0003677	DNA binding	1	Zm00001eb082590
GO:0002229	defense response to oomycetes	1	Zm00001eb167520
GO:0000981	DNA-binding transcription factor activity, RNA polymerase II-specific	1	Zm00001eb117810
GO:0000976	transcription cis-regulatory region binding	1	Zm00001eb179000
GO:0000407	phagophore assembly site	1	Zm00001eb082590
GO:0000373	Group II intron splicing	1	Zm00001eb178330
GO:0000166	nucleotide binding	1	Zm00001eb079800
GO:0000145	exocyst	1	Zm00001eb060520
GO:0000045	autophagosome assembly	1	Zm00001eb036550
GO:2001070	starch binding	1	Zm00001eb070240
